# Effects of psychosocial interventions on cancer-related fatigue in patients with colorectal cancer: a systematic review and meta-analysis of randomised controlled trials

**DOI:** 10.1007/s00520-026-10565-6

**Published:** 2026-03-17

**Authors:** Jinling Lu, Ruohan Wang, Yuen Yu Chong

**Affiliations:** The Nethersole School of Nursing, Faculty of Medicine, The Chinese University of Hong , New Territories, Room 828, Esther Lee Building, Hong Kong SAR, China

**Keywords:** Colorectal cancer, Psychosocial, Cancer-related fatigue, Systematic review

## Abstract

**Purpose:**

This review aimed to scrutinise and critically appraise the evidence on the effects of psychosocial interventions in alleviating CRF for colorectal cancer patients.

**Methods:**

MEDLINE, EMBASE, Cochrane Central Register of Controlled Trials, Web of Science, CINAHL Ultimate, APA PsycInfo, CNKI, and WANFANG Database were electronically searched from inception to 31st August 2025 for randomised controlled trials examining psychosocial interventions for CRF in colorectal cancer patients. Meta-analyses were performed for short-term (immediately post-intervention to 1 month), medium-term (> 1 to 3 months), and long-term (> 3 months) follow-up periods. The certainty of evidence was evaluated using the Grading of Recommendations Assessment, Development and Evaluation (GRADE) approach. The Review Manager Software (Version 5.4.1) was used for data analysis.

**Results:**

Nine studies with 1426 participants were included. Interventions were categorised as psychotherapies, psycho-behavioural interventions, and yoga. Meta-analyses indicated that compared to controls, psychosocial interventions significantly reduced CRF at short-term (standardised mean difference (SMD) = −0.53, 95% confidence interval (CI) = −0.82 to −0.23), medium-term (SMD = −0.51, 95%CI = −0.73 to −0.29), and long-term (SMD = −0.24, 95%CI = −0.46 to −0.01) follow-up. Subgroup analyses indicated that psycho-behavioural interventions were effective (SMD = −0.39, 95% CI = −0.76 to −0.01), while psychotherapy and yoga showed no significant effects. The certainty of evidence ranged from very low to moderate.

**Conclusions:**

Psychosocial interventions, particularly psycho-behavioural approaches, appear effective in reducing CRF among patients with colorectal cancer; however, effects were consistent at medium-term and long-term follow-up, while short-term findings showed substantial heterogeneity. More rigorous, adequately powered trials are needed to strengthen and extend the current evidence base.

**Supplementary Information:**

The online version contains supplementary material available at 10.1007/s00520-026-10565-6.

## Introduction

Colorectal cancer is the third most common cancer globally, and the second in China [1, 2]. The treatments are mainly surgery, supplemented by chemotherapy and/or radiotherapy [3, 4]. Despite the significant advancements in treatments, 32.4%–79% of colorectal cancer patients frequently endure numerous symptoms arising from cancer or its treatments [5], with cancer-related fatigue (CRF) being one of the most common and severe symptoms [6, 7].

Cancer-related fatigue refers to distressing persistent subjective sense of physical (e.g. bodily weakness, reduced stamina), emotional (e.g. loss of motivation, reduced self-esteem, depressive feelings), and/or cognitive (e.g. difficulties in concentration, attention, memory) tiredness or exhaustion associated with cancer or treatment, which is not proportional to recent activity levels and interferes with usual functioning [8, 9]. Colorectal cancer patients experiencing CRF account for 31.1%–69.7%; moreover, CRF may persist or even worsen without proper management [10, 11]. Anxiety symptoms and depressive symptoms have been reported to be associated with CRF in colorectal cancer patients [12]. Besides, CRF is associated with poorer health-related quality of life [13] and increased risks of all-cause mortality [14]. These findings highlight the need for managing CRF in this population.

Methylphenidate is a primary pharmacological option for tackling CRF [15]. Yet, its use increases the risks of vertigo, nausea, and anorexia [16], and it is not recommended as a first-line treatment [8]. Non-pharmacological interventions offer alternative avenues for managing CRF [17]. Physical exercise has been shown to have enduring effects on significantly reducing CRF [18, 19]. However, barriers such as physical limitations or lack of motivation may hinder adherence for some cancer patients, especially for those at an advanced stage [20]. Additionally, nutraceutical supplements, such as Wisconsin ginseng, guarana, and mistletoe, are not recommended as priority treatments due to their uncertain efficacy [17].

Psychosocial interventions refer to non-pharmacological approaches designed to inform, educate, and increase individuals’ coping capacity with diseases and treatments [21]. Several psychosocial interventions are recommended by clinical guidelines to reduce CRF, such as psychoeducation designed to provide informational and psychological support; cognitive-behavioural therapy designed to correct irrational thoughts, emotions, and behaviours and focus on cognitions; and mindfulness-based stress reduction designed to cultivate present-moment awareness without judgement to reduce stress [8, 17]. These psychosocial interventions address CRF by targeting interconnected thoughts, emotions, and/or behaviours related to CRF [22]. However, these clinical guidelines do not specify the optimal types of psychosocial interventions. Moreover, current systematic reviews have demonstrated the effectiveness of psychosocial interventions on reducing CRF; however, they either focused on specific types of psychosocial interventions, such as cognitive training and social support, for colorectal cancer patients [23], or included broad-ranging cancer patients, with less than 10% of participants being colorectal cancer patients [24], limiting the applicability of those findings to this population. To summarise, current literature lacks a comprehensive scientific synthesis or meta-analysis examining the effects of psychosocial interventions on reducing CRF in colorectal cancer patients. This gap underlines the need for targeted research to inform the development of tailored and effective interventions for this specific patient group.

The present systematic review aimed to scrutinise and critically appraise the effects of psychosocial interventions on CRF, anxiety, and depressive symptoms in colorectal cancer patients, and to identify the optimal features for an effective psychosocial intervention for CRF in this population.

## Methods

This systematic review was performed according to the Preferred Reporting Items for Systematic Reviews and Meta-Analysis statement (PRISMA) [25]. The protocol of this review is available on the International Prospective Register of Systematic Reviews (PROSPERO registration number: CRD42024625716).

### Search strategy

MEDLINE, EMBASE, Cochrane Central Register of Controlled Trials, Web of Science, CINAHL Ultimate, APA PsycInfo, CNKI, and WANFANG Database were electronically searched from their inception until 31 st August 2025. WHO International Clinical Trials Registry Platform and ClinicalTrials.gov were searched for grey literature. The reference lists of included articles were manually searched. The Medical Subject Headings (MeSH) terms, along with free-text terms, were used in alignment with the ‘PICOS’ framework: ‘P’-population, ‘I’-intervention, ‘C’-comparison, ‘O’-outcomes, and ‘S’-study design. Keywords included ‘colorectal neoplasms’, ‘psychosocial intervention’, and ‘fatigue’. Details are shown in Supplementary Material A.

### Eligibility criteria

The review included peer-reviewed articles on psychosocial interventions for cancer-related fatigue in patients with colorectal cancer and was restricted to studies published in English or Chinese because of practical constraints related to translation and resource availability. Eligibility criteria were developed according to the PICOS framework and were as follow. 

#### Population

Participants should be individuals aged 18 years or above with a clinical diagnosis of colorectal cancer reported by healthcare professionals. Studies should encompass at least 50% of participants with colorectal cancer [26].

#### Intervention

Interventions should be psychosocial interventions that use psychoeducational, psychotherapeutic, psychological, or behavioural techniques and/or social components to modify individuals’ cognitions, emotions, behaviours, social interactions, or a combination of them [17, 27]. Interventions could be, but are not limited to, psychoeducation, psychotherapy, behavioural therapy, cognitive therapy, cognitive-behavioural therapy, and counselling [21, 27].

#### Comparison

Studies with placebo control, usual care control, no-intervention control, wait-list control, or any active intervention were considered eligible.

#### Outcome

The primary outcome of this review was CRF; therefore, eligible studies were required to focus on CRF. Secondary outcomes were anxiety and depressive symptoms. To be included, studies had to assess CRF as an outcome, with or without concurrent assessment of anxiety and depressive symptoms, and all outcomes had to be measured using validated instruments with published psychometric properties demonstrating acceptable validity and reliability in cancer patients and in the relevant language.

#### Study design

The review exclusively included randomised controlled trials (RCTs). Studies were excluded if they were ongoing studies, comments, protocols, conference abstracts, case reports, book chapters, editorials, or articles with unavailable data.

### Study selection and data extraction

EndNote 20 was initially utilised to automatically merge and remove duplicate articles. Subsequently, two independent reviewers (LJL and WRH) screened the titles and abstracts of the retrieved articles, followed by their independent assessment of the full texts of potentially eligible articles. The data extraction was performed independently by two reviewers (LJL and WRH) with a standardised form, including the first author, publication year, study region, characteristics of participants, characteristics of interventions, control group information, outcomes, data collection time, attrition rate, and effect sizes. Any disagreements were solved by a third reviewer (YYC).

### Study appraisal

The methodological quality of included RCTs was assessed independently by two reviewers (LJL and WRH) with the revised Cochrane risk-of-bias tool for randomised trials (RoB2). The risk of bias of each study was assessed from five domains, including the randomisation process, deviations from intended interventions, missing outcome data, measurement of the outcome, and selection of the reported result [28]. The risk of bias could be determined as ‘Low risk of bias’, ‘Some concerns’, or ‘High risk of bias’. Any disagreements were solved by a third reviewer (YYC).

### Certainty of evidence

The certainty of evidence was assessed independently by two reviewers (LJL and WRH) using the Grading of Recommendations Assessment, Development and Evaluation (GRADE) approach [29]. Six domains were considered: study design, risk of bias, imprecision, inconsistency, indirectness, and magnitude of effect. The certainty of evidence was rated as ‘High’, ‘Moderate’, ‘Low’, or ‘Very low’. Disagreements were resolved by a third reviewer (YYC).

### Data synthesis and analysis

The Review Manager Software (Version 5.4.1) was used for data analysis. Between-group effect sizes were estimated using Cohen’s *d*, calculated from post-intervention scores, aligning with standard practice for randomised controlled trials [30]. Values were obtained either by extracting directly from the included studies or by using the formula:$\mathcal{d}$ = ${(M}_{1}-{M}_{2})/{SD}_{pooled}$, ${SD}_{pooled}$= $\surd [({SD}_{1}^{2}+ {SD}_{2}^{2})/2]$, where *M*_1_, *M*_2_, *SD*_1_, and *SD*_2_ denote the means and standard deviations of the intervention and control groups at each time point, respectively [31]. Cohen’s *d* was chosen due to the moderate-to-large sample size in the included studies (all exceeding 20 participants), under which condition it appropriates Hedges’ *g* closely, minimising small-sample bias [32]. In cases where means and *SD*s were unavailable, effect sizes were derived from reported *t*, *F*, and *P* values.

Data were pooled by follow-up duration: short-term (immediately post-intervention to 1 month), medium-term (> 1 to 3 months), and long-term (> 3 months). Due to substantial heterogeneity in treatments, intervention components, and outcome measures across studies, a random-effects model was used to provide more conservative estimates [33]. Standardised mean differences (SMDs) with 95% confidence intervals (CIs) were utilised to present the pooled effect sizes when outcomes were measured by different instruments, while mean differences (MDs) with 95% CIs were used when outcomes were measured by the same instruments [34]. Statistical significance was considered at* P* < 0.05. Effect sizes of 0.2, 0.5, and 0.8 were considered small, moderate, and large, respectively [31]. The *I*^2^ values were calculated to assess the heterogeneity of the included studies, with values of 0%–40%, 30%–60%, 50%–90%, and 75%–100% indicating unimportant, moderate, substantial, and considerable heterogeneity, respectively [35]. Subgroup analysis based on intervention approaches was undertaken to examine the sources of heterogeneity. In instances where meta-analysis was not feasible due to only one study reporting a given outcome, a narrative synthesis of the findings was conducted following the synthesis without meta-analysis (SWiM) reporting guidelines where applicable [36].

## Results

### Study selection

**Fig. 1:**
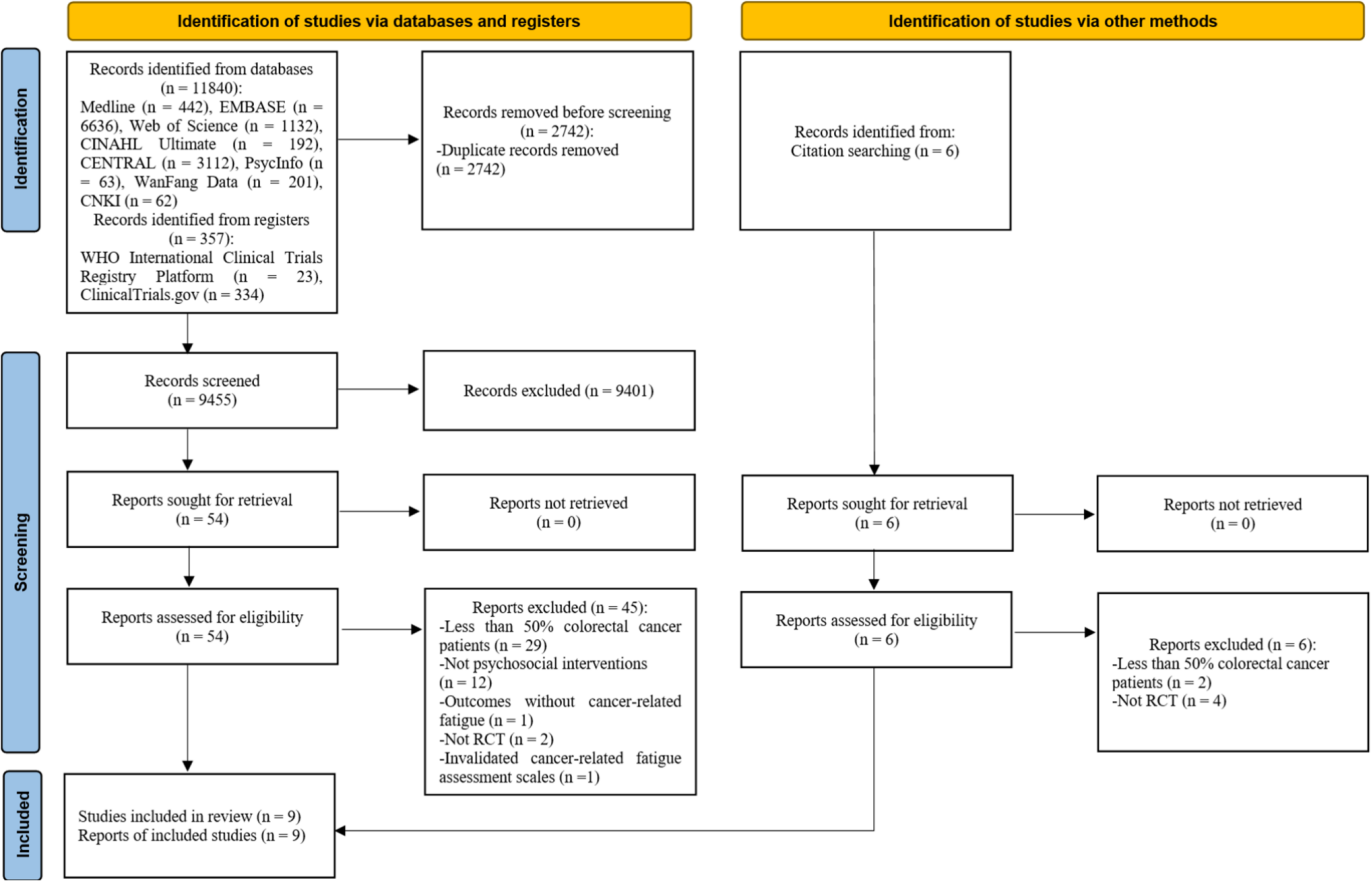
PRISMA flow diagram for study selection

A total of 12,197 records were identified through database and register searches. After removal of duplicates, 9455 records were screened by title and abstract, and 54 articles were assessed in full text. Of these, 45 were excluded because they enrolled fewer than 50% of patients with colorectal cancer, did not evaluate a psychosocial intervention, did not include cancer‑related fatigue as an outcome, were not RCTs, or used non‑validated measures of cancer‑related fatigue. An additional six records identified through citation searching were assessed in full text and excluded because they enrolled fewer than 50% of patients with colorectal cancer or were not RCTs. In total, nine articles reporting nine studies were included in this review. The PRISMA flow diagram of study selection is shown in Fig. 1.

### Characteristics of the included studies

The included nine articles were published from 2013 to 2023. Five studies were conducted in China [37–41], one each in Iran [42], Germany [43], the USA [44], and Australia [45]. The characteristics of the included studies are summarised in Table 1.

**Table 1:** Characteristics of included studies

**First author, year, region**	**Participants**	**Intervention**	**Control**	**Outcomes (measurements)**	**Data collection**	**Attrition rate, between-group effect sizes (Cohen’s *d* (95%CI))**
Lin et al., 2020, China [37]	**Diagnosis, cancer stage** 1. Diagnosis Colon cancer 2. Cancer stage 57% stage Ⅱ, 32% stage Ⅲ, 11% stage Ⅳ **Number of participants per arm (CG vs. IG), age (Mean ± SD), male (%)** 1. Number of participants per arm 100 vs. 100 2. Age No information about average age but patients aged 60 and above accounted for 52% 3. Male 66% **Treatments received/ongoing at enrolment** Had received surgery	**Theoretical basis** Attention and interpretation therapy (AIT) **Content** AIT practices **Approach** Psychotherapy **Format, delivery mode** Group, face to face + telephone **Dosage** Ten weeks **Intervention provider** Nurses	Usual care	**CRF** (PFS-R) **Anxiety symptoms** (SAS) **Depressive symptoms** (SDS)	Two, -Baseline (T1) -Immediately post-intervention (T2)	**T2, 0%** CRF −0.57 (−0.85, −0.28) Anxiety symptoms −3.07 (−3.48, −2.66) Depressive symptoms −0.98 (−1.27, −0.68)
Wang et al., 2023, China [38]	**Diagnosis, cancer stage** 1. Diagnosis Colorectal cancer 2. Cancer stage 25.4% stage Ⅰ, 74.6% stage Ⅱ **Number of participants per arm (CG vs. IG), age (Mean ± SD), male (%)** 1. Number of participants per arm 72 vs. 70 2. Age 62.3 ± 11.7 3.Male 59.2% **Treatments received/ongoing at enrolment** Had completed treatments (e.g. surgery, chemotherapy, radiotherapy)	**Theoretical basis** Not mentioned **Content** Acceptance; behavioural techniques (e.g. information provision, goal setting, action planning, activity monitoring, and problem solving) to promote modifications in physical activity and dietary behaviour **Approach** Psycho-behavioural intervention **Format, delivery mode** Individual, face to face + telephone **Dosage** Fourteen weekly 60-min sessions for 14 weeks **Intervention provider** Nurses	Usual care	**CRF** (FACIT-F) **Anxiety symptoms** (HADS) **Depressive symptoms** (HADS)	Three, -Baseline (T1) -Two weeks post-intervention (T2) -Ten weeks post-intervention (T3)	**T2, 6.4%** CRF −0.27 (−0.60, 0.06) Anxiety symptoms −0.03 (−0.36, 0.30) Depressive symptoms −0.28 (−0.61, 0.05) **T3, 9.0%** CRF −0.33 (−0.66, 0.01) Anxiety symptoms −0.14 (−0.47, 0.19) Depressive symptoms −0.19 (−0.52, 0.14)
Xian et al., 2022, China [39]	**Diagnosis, cancer stage** 1. Diagnosis Colorectal cancer 2. Cancer stage 21% stage Ⅱ, 58.8% stage Ⅲ, 20.2% stage Ⅳ **Number of participants per arm (CG vs. IG), age (Mean ± SD), male (%)** 1. Number of participants per arm 59 vs. 60 2. Age 60.7 ± 10.8 3. Male 58.5% **Treatments received/ongoing at enrolment** During chemotherapy	**Theoretical basis** Solution-focused therapy (SFT) **Content** SFT practices **Approach** Psychotherapy **Format, delivery mode** Individual, face to face **Dosage** Six monthly 30-min sessions for 6 months **Intervention provider** Nurses	Health education	**CRF** (CFS)	Three, -Baseline (T1) -Three months post-intervention (T2) -Six months post-intervention (T3)	**T2, 4.0%** CRF −0.84 (−1.21, −0.46) **T3, 16.1%** CRF −0.41 (−0.77, −0.05)
Xie et al., 2020, China [40]	**Diagnosis, cancer stage** 1. Diagnosis 79.1% colorectal cancer, 20.9% stomach cancer 2. Cancer stage 18.6% stage Ⅱ, 50% stage Ⅲ, 31.4% stage Ⅳ **Number of participants per arm (CG vs. IG), age (Mean ± SD), male (%)** 1. Number of participants per arm 42 vs. 44 2. Age -CG: 52.9 ± 10.4 -IG: 53.4 ± 9.7 3. Male 51.2% **Treatments received/ongoing at enrolment** During chemotherapy	**Theoretical basis** Not mentioned **Content** Emotion expression; behavioural techniques (e.g. information provision) to promote modifications in dietary behaviour **Approach** Psycho-behavioural intervention **Format, delivery mode** Not mentioned, face to face **Dosage** Twelve weekly 3-min sessions for 12 weeks **Intervention provider** Nurses	Usual care	**CRF** (CFS)	Two, -Baseline (T1) -Immediately post-intervention (T2)	**T2, 5.5%** CRF −0.32 (−0.74, 0.11)
Chen et al., 2019, China [41]	**Diagnosis, cancer stage** 1. Diagnosis 51.1% colorectal cancer, 48.9% stomach cancer 2. Cancer stage 21.2% stage Ⅰ, 52.3% stage Ⅱ, 26.5% stage Ⅲ **Number of participants per arm (CG vs. IG), age (Mean ± SD), male (%)** 1. Number of participants per arm 161 vs.160 2. Age No information about average age but patients aged 60 and above accounted for 55.5% 3. Male 67.3% **Treatments received/ongoing at enrolment** During chemotherapy	**Theoretical basis** Not mentioned **Content** Emotion expression; behavioural techniques (e.g. information provision, problem solving) to promote modifications in physical activity, dietary behaviour, and sleep hygiene **Approach** Psycho-behavioural intervention **Format, delivery mode** Group, face to face + telephone **Dosage** Six weekly 30-min sessions for 6 weeks **Intervention provider** Nurses	Usual care	**CRF** (CFS)	Two, -Baseline (T1) -Immediately post-intervention (T2)	**T2, 0%** CRF −0.83 (−1.05, −0.60)
Yousefi et al., 2022, Iran [42]	**Diagnosis, cancer stage** 1. Diagnosis 62% colorectal cancer, 38% stomach cancer 2. Cancer stage Encompassing stage Ⅰ, Ⅱ, Ⅲ, but no information about the percentage **Number of participants per arm (CG vs. IG), age (Mean ± SD), male (%)** 1. Number of participants per arm 25 vs. 25 2. Age 54.9 ± 6.6 3. Male 58% **Treatments received/ongoing at enrolment** During chemotherapy	**Theoretical basis** Mindfulness-based stress reduction (MBSR) **Content** MBSR practices **Approach** Psychotherapy **Format, delivery mode** Individual, internet **Dosage** Nine weekly 90-min sessions for 9 weeks **Intervention provider** Not mentioned	Usual care	**CRF** (fatigue subscale of EORTC QLQ-C30)	Three, -Baseline (T1) -Immediately post-intervention (T2) -Two months post-intervention (T3)	**T2, 6%** CRF −1.82 (−2.49, −1.16) **T3, 10%** CRF −0.60 (−1.16, −0.03)
Cramer et al., 2016, Germany [43]	**Diagnosis, cancer stage** 1. Diagnosis Colorectal cancer 2. Cancer stage 37.0% stage Ⅰ, 20.4% stage Ⅱ, 38.9% stage Ⅲ, 3.7% stage Ⅳ **Number of participants per arm (CG vs. IG), age (Mean ± SD), male (%)** 1. Number of participants per arm 27 vs. 27 2. Age 68.3 ± 9.7 3. Male 61.1% **Treatments received/ongoing at enrolment** During chemotherapy	**Theoretical basis** Not mentioned **Content** Yoga practices **Approach** Yoga **Format, delivery mode** Group, NA **Dosage** Ten weekly 90-min sessions for 10 weeks **Intervention provider** Yoga instructors	Wait-list control	**CRF** (FACIT-F) **Anxiety symptoms** (HADS) **Depressive symptoms** (HADS)	Three, -Baseline (T1) -Immediately post-intervention (T2) -Three months post-intervention (T3)	**T2, 20.4%** CRF −0.35 (−0.88, 0.19) Anxiety symptom −0.64 (−1.19, −0.09) Depressive symptom −0.59 (−1.13, −0.04) **T3, 20.4%** CRF −0.35 (−0.89, 0.19) Anxiety symptom −0.25 (−0.78, 0.29) Depressive symptom −0.38 (−0.91, 0.16)
Sohl et al., 2022, USA [44]	**Diagnosis, cancer stage** 1. Diagnosis Colorectal cancer 2. Cancer stage 14% stage Ⅱ, 42% stage Ⅲ, 44% stage Ⅳ **Number of participants per arm (CG vs. IG), age (Mean ± SD), male (%)** 1. Number of participants per arm 21 vs. 23 2. Age 58.5 ± 11.7 3. Male 48% **Treatments received/ongoing at enrolment** During chemotherapy	**Theoretical basis** Not mentioned **Content** Yoga practices **Approach** Yoga **Format, delivery mode** Individual, face to face **Dosage** Four biweekly 30-min sessions for 8 weeks **Intervention provider** Yoga instructors	Attention control	**CRF** (PROMIS fatigue instrument) **Depressive symptom** (PROMIS depression instrument)	Three, -Baseline (T1) -Two weeks post-intervention (T2) -Six weeks post-intervention (T3)	**T2, 18.2%** CRF −0.31 (−0.90, 0.29) Depressive symptom −0.11 (−0.71, 0.48) **T3, 29.5%** CRF −0.40 (−0.99, 0.20) Depressive symptom −0.42 (−1.02, 0.17)
Hawkes et al., 2013, Australia [45]	**Diagnosis, cancer stage** 1. Diagnosis Colorectal cancer 2. Cancer stage 18.3% stage Ⅰ, 28.8% stage Ⅱ, 22.7% stage Ⅲ, 30.2% unknown **Number of participants per arm (CG vs. IG), age (Mean ± SD), male (%)** 1. Number of participants per arm 205 vs. 205 2. Age -CG: 67.8 ± 9.2 -IG: 64.9 ± 10.8 3. Male 53.9% **Treatments received/ongoing at enrolment** Not mentioned	**Theoretical basis** Not mentioned **Content** Acceptance; behavioural techniques (e.g. information provision, goal setting, action planning, activity monitoring, and problem solving) to promote modifications in physical activity and dietary behaviour **Approach** Psycho-behavioural intervention **Format, delivery mode** Individual, telephone **Dosage** Twelve biweekly sessions for 6 months **Intervention provider** Health coaches	Usual care	**CRF** (FACIT-F)	Three, -Baseline (T1) -Immediately post-intervention (T2) -Six months post-intervention (T3)	**T2, 15.4%** CRF −0.12 (−0.31, 0.08) **T3, 21.5%** CRF −0.16 (−0.35, 0.04)

*Notes.* *CFS*, Cancer Fatigue Scale; *CG*, control group; *CI*, confidence interval; *CRF*, cancer-related fatigue; *EORTC QLQ*, European Organization for Research and Treatment of Cancer Core Quality of Life Questionnaire; *FACIT-F*, Functional Assessment of Chronic Illness Therapy-Fatigue; *HADS*, Hospital Anxiety and Depression Scale; *IG*, intervention group; *PFS-R*, Revised Piper Fatigue Scale; *PROMIS*, Patient Reported Outcomes Measurement Information System; *SAS*, Self-Rating Anxiety Scale; *SD*, standard deviation; *SDS*, Self-Rating Depression Scale; *T1*, Time 1; *T2*, Time 2; *T3*, Time 3

#### Characteristics of participants

A total of 1426 colorectal cancer patients (59% male) were included. The sample size ranged from 44 to 410 per study, and the mean age (standard deviation) of the participants ranged from 52.9 (10.4) to 68.3 (9.7) years. Six studies only included colorectal cancer patients [37–39, 43–45]. The remaining three studies primarily (> 50%) focused on colorectal cancer patients [40–42]. Most studies (8/9) included patients across stages I–IV [37, 39–45], while one focused only on those with stages I–II [38]. With respect to treatment status, six studies enrolled patients undergoing chemotherapy [39–44], one post-surgery [37], one after treatment completion [38], and one did not specify [38, 45].

#### Characteristics of interventions

Given the substantial overlap in psychological and behavioural components across trials, interventions were categorised according to their predominant modality and theoretical orientation rather than a strict psychological-versus-behavioural dichotomy. This approach acknowledges that many interventions, including yoga, integrate elements of psychological support, behaviour change, and exercise, and are therefore inherently multicomponent. Although this classification is partially aligned with commonly used subgroupings in the literature (e.g. psychological support, behavioural interventions, exercise interventions, social support, multicomponent interventions), finer disaggregation was not feasible because of component overlap and the small number of trials, which would have yielded small, heterogeneous subgroups with limited interpretability. Accordingly, interventions were grouped into three categories: psycho-behavioural interventions, psychotherapies, and yoga.

*Psycho-behavioural interventions* (four studies [38, 40, 41, 45]) combined psychological strategies (e.g. emotional expression, acceptance-based techniques, psychoeducation) with behavioural techniques targeting health-related behaviours [46]. Emotional expression facilitated recognition and externalisation of suppressed emotions in two studies [40, 41], whereas acceptance-based strategies encouraged non-judgmental engagement with CRF in two others [38, 45]. All four included studies incorporated behavioural elements, including information provision, goal setting, action planning, problem-solving, and activity monitoring, to modify physical activity, diet behaviours, and sleep hygiene [38, 40, 41, 45].

*Psychotherapies* (three studies [37, 39, 42]) adhered to established psychotherapeutic models with structured protocols and explicit theoretical frameworks. Attention and Interpretation Therapy promoted present-moment awareness and reinterpretation of CRF experiences through gratitude, compassion, acceptance, meaning, and forgiveness [37]; Solution-Focused Therapy leveraged patients’ past successes and strengths to support solution-building [39]; and Mindfulness-Based Stress Reduction used mindfulness practices to mitigate stress and enhance coping with CRF [42].

Yoga (two studies [43, 44]) emphasised structured postures, breathing exercises, and mindfulness to foster mind–body integration and relaxation, incorporating psychological-behavioural elements such as stress reduction and self-regulation.

Across all categories, interventions were delivered individually (5/9 studies [38, 39, 42, 44, 45]), in groups (3/9 [37, 41, 43]), or with unspecified format (1/9 [40]). Delivery modes included blended telephone and face-to-face (3/9 [37, 38, 41]), face-to-face only (3/9 [39, 40, 44]), internet-based (1/9) [42], telephone only (1/9) [45], or unspecified (1/9) [43]. Sessions typically numbered 6–14, lasted 60–90 min, and were delivered weekly, biweekly, or monthly over 6 weeks to 6 months. Providers were nurses (5/9) [37–41], yoga instructors (2/9) [43, 44], health coaches (1/9) [45], or unspecified (1/9) [42].

#### Characteristics of comparisons

The comparisons were typically usual care (6/9) [37, 38, 40–42, 45], and the others involved health education [39], attention control [44], and wait-list control [43].

#### Outcome measures and instruments

Cancer-related fatigue (CRF) was measured in all studies using the Cancer Fatigue Scale (CFS) [39–41], Revised Piper Fatigue Scale (PFS-R) [37], Functional Assessment of Chronic Illness Therapy-Fatigue (FACIT-F) [38, 43, 45], European Organization for Research and Treatment of Cancer Core Quality of Life Questionnaire (EORTC QLQ) fatigue subscale [42], and the Patient Reported Outcomes Measurement Information System (PROMIS) fatigue instrument [44]. Anxiety was measured in three studies using the Hospital Anxiety and Depression Scale (HADS) [38, 43] and the Self-Rating Anxiety Scale (SAS) [37]. Depression was measured in four studies using HADS [38, 43], the Self-Rating Depression Scale (SDS) [37], and the PROMIS depression instrument [44].

#### Attrition

Attrition rates ranged from 0% to 20.4% at short-term follow-up, from 4% to 29.5% at medium-term follow-up, and from 16.1% to 21.5% at long-term follow-up. Notably, two studies [37, 41] reported an attrition rate of 0% at short-term follow-up; these were conducted in hospital settings or involved brief intervention durations, potentially facilitating complete participant retention during the study period.

### Methodological quality of the included studies

**Fig. 2:**
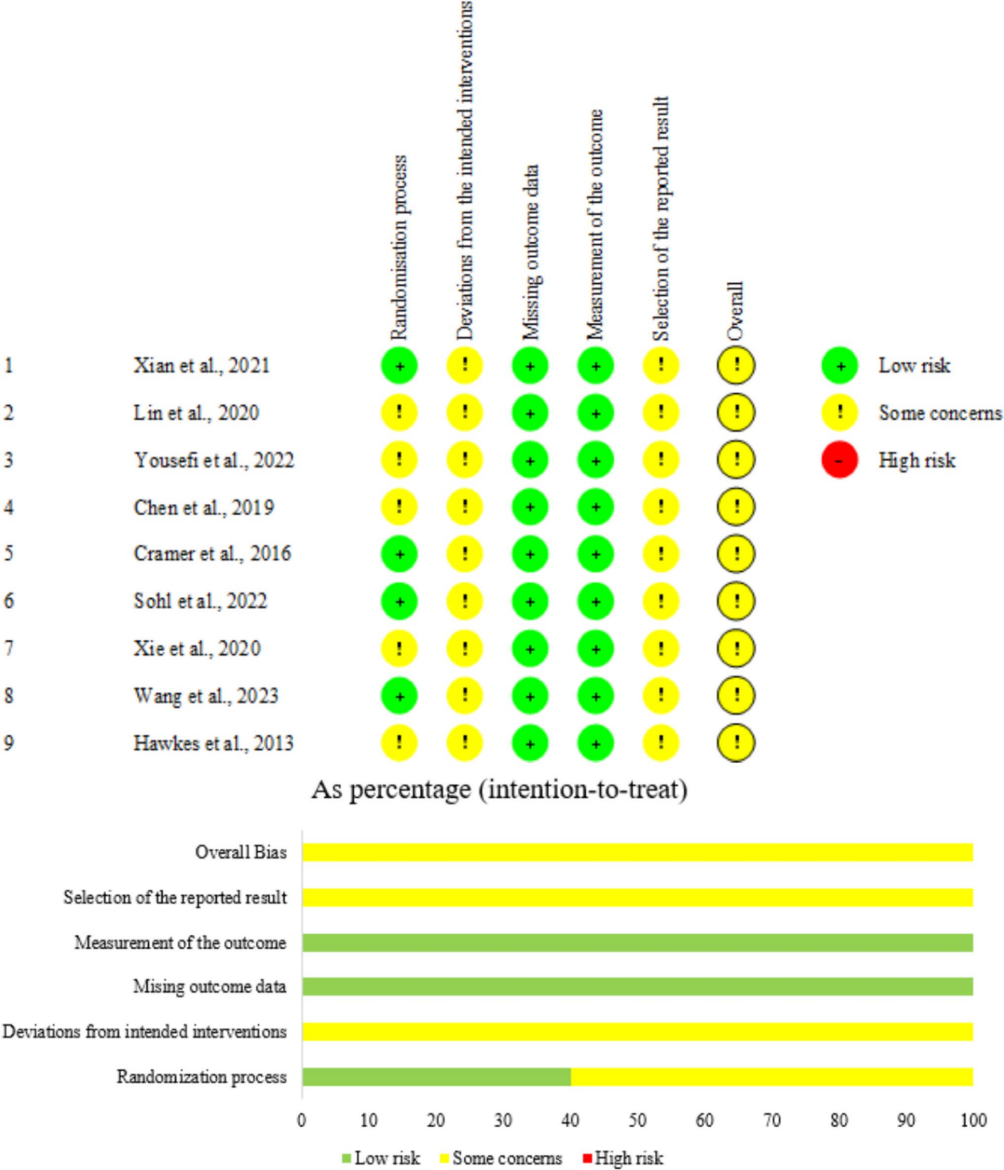
Summary of risk of bias for the included studies

The quality of nine RCTs was considered with some concerns. Most concerns arose from insufficient information on allocation concealment, the effect of assignment to intervention, and pre-specified analysis plans. Detailed results are shown in Fig. 2.

### Intervention effects on cancer-related fatigue

Eight studies assessed CRF at short-term follow-up [37, 38, 40–45], five examined CRF at medium-term follow-up [38, 39, 42–44], and two examined CRF at long-term follow-up [39, 45].

#### Effects at short-term follow-up

**Fig. 3:**
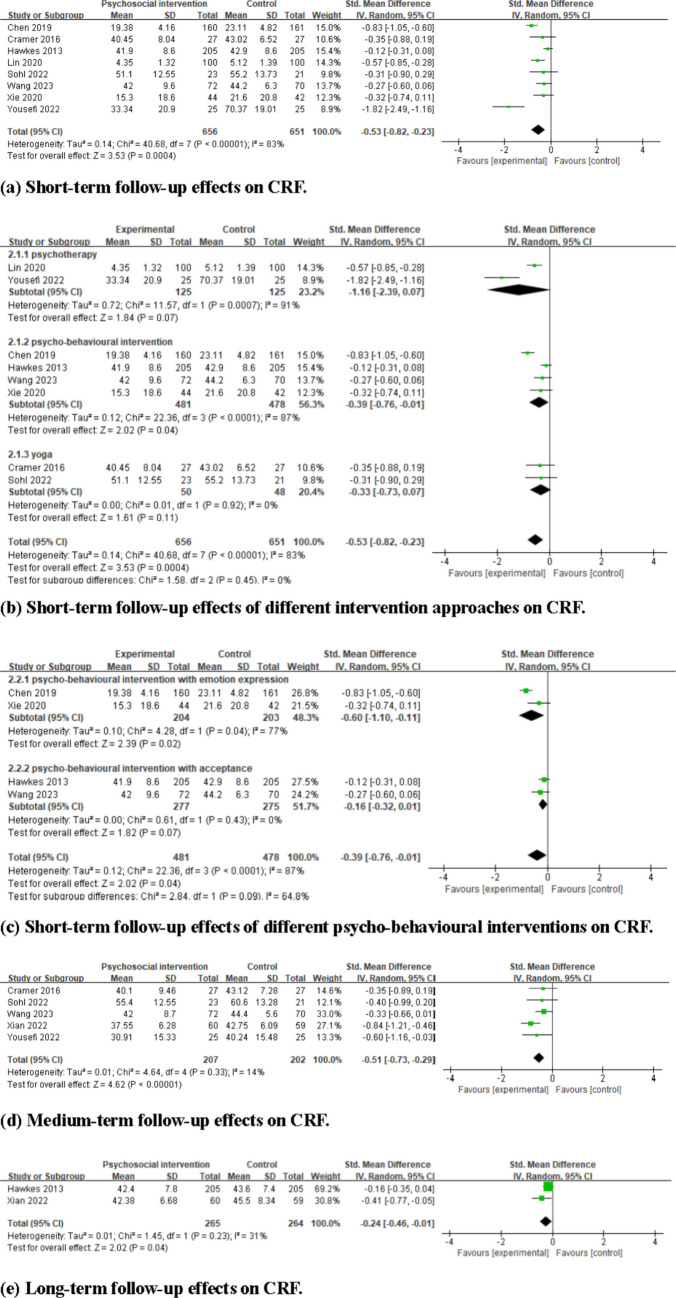
Forest plots of the effects of psychosocial interventions on cancer-related fatigue (CRF). **a** Short-term follow-up effects on CRF. **b** Short-term follow-up effects of different intervention approaches on CRF. **c** Short-term follow-up effects of different psycho-behavioural interventions on CRF. **d** Medium-term follow-up effects on CRF. **e** Long-term follow-up effects on CRF

The pooled results of eight studies indicated that when compared to the control group, psychosocial interventions significantly reduced CRF at short-term follow-up (SMD = −0.53, 95% CI = −0.82 to −0.23, *I*^2^ = 83%, 8 studies, 1307 participants), as shown in Fig. 3a. Subgroup analysis indicated that when compared to the control group, psycho-behavioural intervention significantly reduced CRF (SMD = −0.39, 95% CI = −0.76 to −0.01, *I*^2^ = 87%, 4 studies, 959 participants); however, no significant effects were observed for psychotherapy (SMD = −1.16, 95% CI = −2.39 to 0.07, *I*^2^ = 91%, 2 studies, 250 participants) and yoga (SMD = −0.33, 95% CI = −0.73 to 0.07, *I*^2^ = 0%, 2 studies, 98 participants), as shown in Fig. 3b. Subgroup analysis also indicated that psycho-behavioural intervention incorporating emotion expression significantly reduced CRF compared to the control group (SMD = −0.60, 95% CI = −1.10 to −0.11, *I*^2^ = 77%, 2 studies, 407 participants), whereas those incorporating acceptance did not demonstrate a significant effect (SMD = −0.16, 95% CI = −0.32 to 0.01, *I*^2^ = 0%, 2 studies, 552 participants), as shown in Fig. 3c. Notably, these pooled effect sizes displayed substantial heterogeneity, with most *I*^2^ exceeding 75%, regardless of the subgroup analysis conducted.

#### Effects at medium-term follow-up

The pooled result of five studies showed that when compared to the control group, psychosocial interventions significantly reduced CRF at medium-term follow-up (SMD = −0.51, 95% CI = −0.73 to −0.29, *I*^2^ = 14%, 5 studies, 409 participants) (see Fig. 3d).

#### Effects at long-term follow-up

The pooled result of two studies indicated that when compared to the control group, psychosocial interventions significantly reduced CRF at long-term follow-up (SMD = −0.24, 95% CI = −0.46 to −0.01, *I*^2^ = 31%, 2 studies, 529 participants), as shown in Fig. 3e.

### Intervention effects on anxiety symptoms

Three studies assessed anxiety symptoms at short-term follow-up [37, 38, 43]; two examined anxiety symptoms at medium-term follow-up [38, 43]. No study reported long-term outcomes for anxiety symptoms.

#### Effects at short-term follow-up

**Fig. 4:**
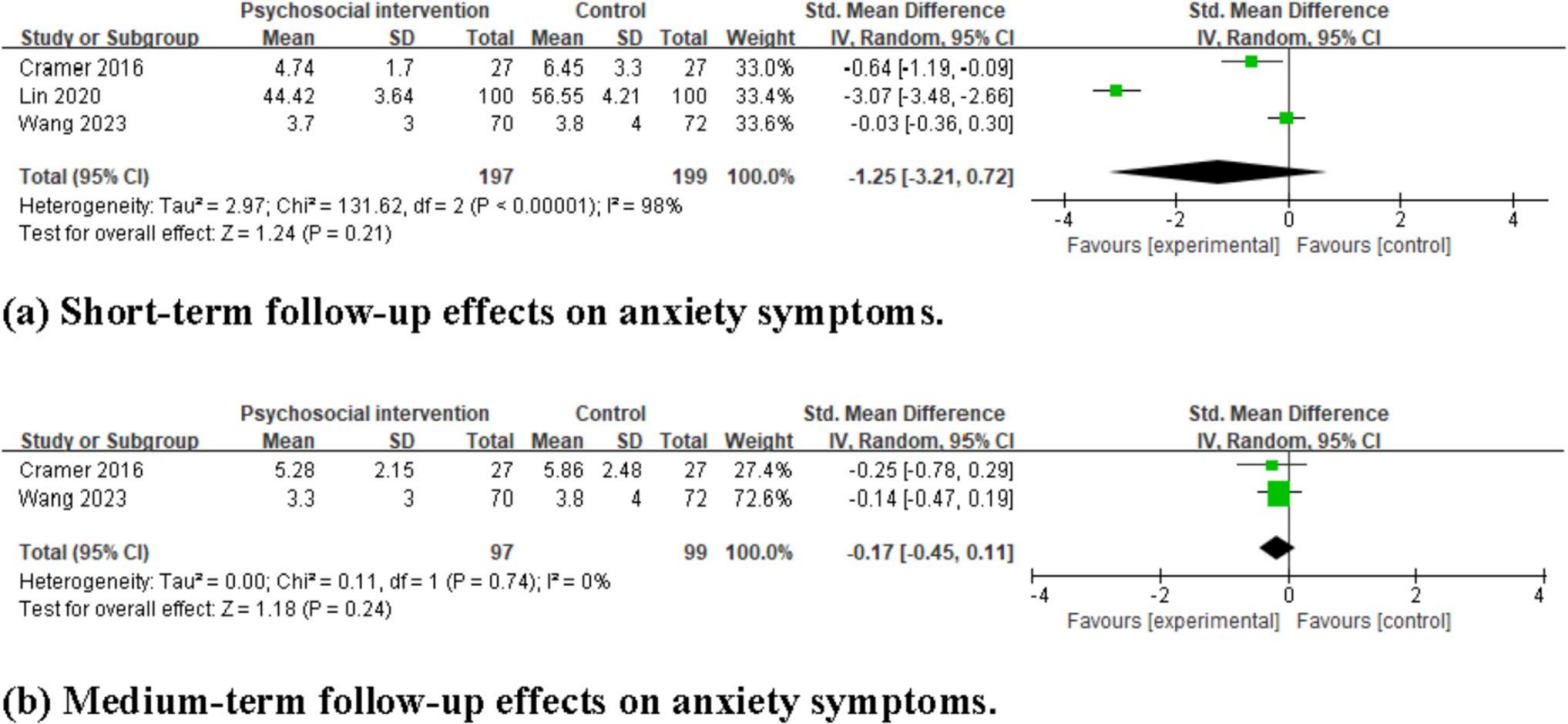
Forest plots for the effects of psychosocial interventions on anxiety symptoms. **a** Short-term follow-up effects on anxiety symptoms. **b** Medium-term follow-up effects on anxiety symptoms

The pooled results of three studies show that psychosocial intervention did not significantly reduce anxiety symptoms at short-term follow-up compared to the control group (SMD = −1.25, 95% CI = −3.21 to 0.72, *I*^2^ = 98%, 3 studies, 396 participants), as shown in Fig. 4a**.**

#### Effects at medium-term follow-up

The pooled results of two studies indicated that psychosocial interventions did not significantly reduce anxiety symptoms at medium-term follow-up compared to the control group (SMD = −0.17, 95%CI = −0.45 to 0.11, *I*^2^ = 0%, 2 studies, 196 participants) (see Fig. 4b).

### Intervention effects on depressive symptoms

Four studies assessed depressive symptoms at short-term follow-up [37, 38, 43, 44], and three examined depressive symptoms at medium-term follow-up [38, 43, 44], while no study reported long-term outcomes for depressive symptoms.

#### Effects at short-term follow-up

**Fig. 5:**
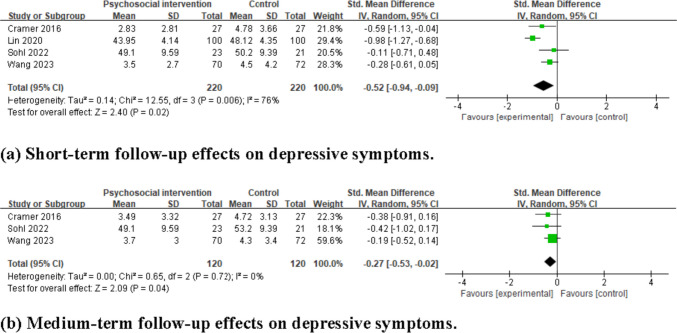
Forest plots for the effects of psychosocial interventions on depressive symptoms. **a** Short-term follow-up effects on depressive symptoms. **b** Medium-term follow-up effects on depressive symptoms

The pooled results of four studies showed that psychosocial interventions significantly reduced depressive symptoms at short-term follow-up compared to the control group (SMD = −0.52, 95%CI = −0.94 to −0.09, *I*^2^ = 76%, 4 studies, 440 participants), as shown in Fig. 5a.

#### Effects at medium-term follow-up

The pooled results of three studies indicated that psychosocial interventions significantly reduced depressive symptoms at medium-term follow-up compared to the control group (SMD = −0.27, 95%CI = −0.53 to −0.02, *I*^2^ = 0%, 3 studies, 240 participants), as shown in Fig. 5b.

### Quality of evidence

The certainty of evidence was rated from very low to moderate, as presented in Table 2. The pooled results indicated a serious risk of bias, as all the information is derived from studies with some concerns. Furthermore, the serious inconsistency observed was primarily attributed to the considerable heterogeneity among the included studies. Additionally, the impression of the evidence was deemed to be serious due to the total sample size falling below the recommended threshold of 400 participants.

**Table 2:** Certainty of evidence

**Certainty assessment**						**No. of patients**		**Effects**		**Certainty**	**Importance**
**No. of studies**	**Study design**	**Risk of bias**	**Inconsistency**	**Indirectness**	**Imprecision**	**Intervention**	**Control**	**Relative (95% CI)**	**Absolute (95% CI)**		
Cancer-related fatigue, assessed with the fatigue subscale of EORTC QLQ-C30, CFS, PFS-R, FACIT-F, and PROMIS at short-term follow-up (immediately post-intervention to 1 month)											
8	RCT	Serious ^a^	Serious ^b^	Not serious	Not serious	656	651	NA	SMD = −0.53 95%C*I* = −0.82 to −0.23	⨁⨁◯◯ Low	CRITICAL
Cancer-related fatigue, assessed with EORTC QLQ-C30, CFS, FACIT-F, and PROMIS at medium-term follow-up (> 1 to 3 months)											
5	RCT	Serious ^a^	Not serious	Not serious	Not serious	207	202	NA	SMD = −0.51 95%C*I* = −0.73 to −0.29	⨁⨁⨁◯ Moderate	CRITICAL
Cancer-related fatigue, assessed with EORTC QLQ-C30, CFS, and FACIT-F at long-term follow-up (> 3 months)											
2	RCT	Serious ^a^	Not serious	Not serious	Not serious	265	264	NA	SMD = −0.24 95% CI = −0.46 to −0.01	⨁⨁⨁◯ Moderate	CRITICAL
Anxiety symptom, assessed with SAS and HADS at short-term follow-up (immediately post-intervention to 1 month)											
3	RCT	Serious ^a^	Serious ^c^	Not serious	Serious ^d^	197	199	NA	SMD = −1.25 95%C*I* = −3.21 to 0.72	⨁◯◯◯ Very low	CRITICAL
Anxiety symptom, assessed with HADS at medium-term follow-up (> 1 to 3 months)											
2	RCT	Serious ^a^	Not serious	Not serious	Serious ^d^	97	99	NA	SMD = −0.17 95%CI = −0.45 to 0.11	⨁⨁◯◯ Low	CRITICAL
Depressive symptoms, assessed with SDS, HADS, and PROMIS at short-term follow-up (immediately post-intervention to 1 month)											
4	RCT	Serious ^a^	Serious ^e^	Not serious	Not serious	220	220	NA	SMD = −0.52 95%C*I* = −0.94 to −0.09	⨁⨁◯◯ Low	CRITICAL
Depressive symptoms, assessed with HADS and PROMIS at medium-term follow-up (> 1 to 3 months)											
3	RCT	Serious ^a^	Not serious	Not serious	Serious ^d^	120	120	NA	SMD = −0.27 95%C*I* = −0.53 to −0.02	⨁⨁◯◯ Low	CRITICAL

*Notes.* *CFS*, Cancer Fatigue Scale; *EORTC QLQ-C30*, European Organization for Research and Treatment of Cancer Core Quality of Life Questionnaire; *FACIT-F*, Functional Assessment of Chronic Illness Therapy-Fatigue; *HADS*, Hospital Anxiety and Depression Scale; *NA*, not applicable; *PFS-R*, Revised Piper Fatigue Scale; *PROMIS*, Patient Reported Outcomes Measurement Information System; *SAS*, Self-Rating Anxiety Scale; *SDS*, Self-Rating Depression Scale

^a^All the information is derived from studies with some concerns

^b^Considerable heterogeneity (*I*^2^ = 83%)

^c^Considerable heterogeneity (*I*^2^ = 98%)

^d^The total sample size is less than the recommended number of 400

^e^Considerable heterogeneity (*I*^2^ = 76%)

## Discussion

This systematic review of nine randomised controlled trials (RCTs) evaluated the effects of psychosocial interventions, categorised as psycho-behavioural interventions, psychotherapy, and yoga, on CRF, anxiety symptoms, and depressive symptoms in colorectal cancer patients. The interventions, typically comprising six to 14 sessions of 30 to 90 min each, were mostly delivered individually via telephone or the internet. Meta-analyses showed that psychosocial interventions significantly reduced CRF at short-term (immediately post-intervention to 1 month), medium-term (> 1 to 3 months), and long-term (> 3 months) follow-up, as well as depressive symptoms at short- and medium-term follow-up. No significant effects were observed for anxiety symptoms at any time point. Subgroup analyses indicated significant effects of psycho-behavioural interventions on CRF. Given that the certainty of evidence ranged from very low to moderate, these findings should be interpreted with caution.

A key finding is that psychosocial interventions produce sustained small to moderate reductions in CRF, consistent with prior systematic reviews for breast and lung cancer populations [47, 48]. However, substantial heterogeneity was observed for pooled effects at short-term follow-up (*I*^2^ = 83%), with variability decreasing at medium-term (*I*^2^ = 14%) and long-term (*I*^2^ = 31%) follow-up. The heterogeneous short-term effects may be attributed to high sensitivity to variations in intervention characteristics (e.g. format, delivery mode, dosage), methodological differences, and patient factors (e.g. cancer stage, treatment status) at this time point, which varied across studies. In contrast, medium-term and long-term effects may reflect more stable, sustained benefits that emerge after patients adapt to the intervention or complete treatments.

Subgroup analysis revealed that psycho-behavioural interventions appear particularly promising, with a small-to-moderate effect size shown at short-term follow-up. These interventions integrate psychological techniques (e.g. emotional expression) with behavioural strategies (e.g. information provision, goal setting, action planning, problem-solving, and activity monitoring) to promote changes in physical activity, dietary behaviours, and sleep hygiene. This approach is theoretically grounded in the cognitive-behavioural model of fatigue, which attributes fatigue to maladaptive thoughts and behaviours that maintain physiological dysregulation and functional impairment [49]. On the one hand, the aforementioned behavioural strategies, which systematically deconstructed complex wellness goals into manageable and actionable steps, are used to directly facilitate these three domains, influencing multiple physiological pathways implicated in CRF. Specifically, physical activity can modulate systemic inflammation, improve cardiorespiratory fitness, and enhance mitochondrial function and muscle strength, thereby improving perceived energy and reducing fatigue [8, 17]. Sleep hygiene interventions can help normalise circadian rhythms and restore more consolidated and restorative sleep, lowering levels of inflammatory markers and improving neuroendocrine regulation [50]. Dietary interventions promoting adequate energy, protein, and nutrient intake mitigate treatment-related metabolic disturbances, reduce sarcopenia, and support immune function [8, 17]. On the other hand, in terms of the psychological level, emotional expression and related techniques may reduce emotional suppression and chronic stress arousal, potentially influencing hypothalamic–pituitary–adrenal (HPA) axis activity and autonomic balance [51]. In combination, these behavioural and psychological changes could work synergistically to restore or enhance energy and attenuate fatigue through both direct physiological effects and indirect effects mediated by improved self-regulation and adherence. However, in the two psycho-behavioural studies included in this review, relevant mechanistic variables (e.g. inflammatory markers, objective sleep parameters, physical activity levels, or dietary indicators) were not assessed as outcomes [40, 41]. Future trials should incorporate such mediators to elucidate the physiological and behavioural mechanisms underlying treatment effects.

Regarding the modalities of psychosocial interventions aimed at reducing CRF, an individual approach is the most commonly utilised format. Individual sessions provide personalised attention to each participant, addressing their specific needs and concerns, thereby enhancing the therapeutic experience [52]. Moreover, participants may feel comfortable when discussing sensitive issues related to their diagnosis and treatments in a one-on-one setting, fostering greater openness and honesty—especially for colorectal cancer patients with a stoma who may feel ashamed about their appearance and bowel function [53, 54]. In terms of delivery modes, more than half of the included studies utilised telemedicine such as telephone and internet, either alone or in combination with face-to-face interaction. From the cancer patients’ perspectives, despite physical distance and time constraints, telemedicine enables them to have immediate access to professional guidance, minimising disruption to their lives caused by the disease [55]. A systematic review also indicated that among individuals with mental health issues, no significant differences in efficacy on symptom severity were observed between psychosocial interventions delivered via telemedicine and those delivered face to face [56]. Besides, regarding dosage, findings suggested a minimum of six sessions, with each session lasting 30 min and held weekly. However, limited studies precluded subgroup analysis comparing intervention modalities or dosage for reducing CRF. Consequently, further research is required to determine optimal intervention modalities and dosage for this patient population.

Another observation is that psychosocial intervention could reduce depressive symptoms, demonstrating small to moderate effect sizes at short- and medium-term follow-up. Similar findings have been reported in another systematic review, which indicated significant effects of psychosocial interventions on reducing depressive symptoms in breast cancer patients, with effect sizes ranging from small to moderate [57]. Notably, depressive symptoms and CRF share several overlapping symptom manifestations, such as weakness, decreased energy, diminished concentration and attention, loss of interest, and sadness [58]. These similarities in manifestations contribute significantly to the positive correlation between the two conditions [59], which may elucidate that psychosocial interventions designed to reduce CRF could also alleviate depressive symptoms.

Turning to anxiety symptoms, the effects of psychosocial interventions remained non-significant; however, the certainty of this evidence ranged from very low to low. Furthermore, anxiety symptoms, depressive symptoms, and cancer-related fatigue tend to co-occur as a symptom cluster in patients with colorectal cancer, with potential bidirectional interactions among these symptoms [60]. This complexity underscores the need for further research to draw definitive conclusions about the management of symptom clusters in this population.

### Limitations

To our knowledge, this is the first systematic review and meta-analysis synthesising evidence on psychosocial interventions for CRF in colorectal cancer patients, encompassing related outcomes such as anxiety and depressive symptoms. Several limitations must be acknowledged to contextualise the findings. First, the overall certainty of evidence ranged from very low to moderate, reflecting methodological shortcomings in the included trials (e.g. unclear allocation concealment, incomplete reporting of pre-specified analyses) and limiting confidence in the pooled estimates. Second, the small number of eligible RCTs (*n* = 9) and their heterogeneity, including mixed disease stages (I–IV), predominantly patients receiving chemotherapy, wide variation in intervention content, intensity, duration, and delivery modes, and a scarcity of long-term follow-up, likely contributed to the moderate statistical heterogeneity and restricted the precision and generalisability of the findings. Third, although interventions could conceptually be grouped by functional components (e.g. psychological support, behavioural strategies, exercise, social support, multicomponent), substantial overlap in components precluded robust component-based subgroup analyses; we therefore adopted a modality- and theory-based classification (yoga, psycho-behavioural interventions, psychotherapy), while acknowledging their multicomponent nature and the consequent difficulty in isolating specific effects. Fourth, publication bias could not be formally assessed because fewer than ten studies contributed to each meta-analysis. Finally, only one trial focused on post-treatment survivors, who continue to experience CRF but commonly lack professional support and resources [61], limiting inferences about long-term and post-treatment effectiveness.

### Implications for practice and research

This review provides evidence-based insights into CRF management and underscores the potential benefits of psychosocial interventions, particularly psycho-behavioural approaches that incorporate emotion expression and behavioural techniques (e.g. information provision, goal setting, action planning, activity monitoring, and problem-solving) to promote adaptive changes in physical activity, dietary behaviours, and sleep hygiene, for colorectal cancer patients. Building on the identified limitations, future RCTs should prioritise exploring optimal modalities, dosages, and long-term efficacy, while stratifying by disease stage to enhance efficacy consistency. Investigations of psycho-behavioural interventions should include physical activity, dietary behaviours, and sleep hygiene as mediator outcomes to elucidate mechanisms. Greater attention should be directed towards post-treatment survivors. Lastly, adherence to CONSORT-SPI 2018 guidelines is essential to improve methodological rigour [62]. Addressing these gaps will strengthen the evidence base for informed decision-making in colorectal cancer CRF care.

## Conclusion

This review suggests that psychosocial interventions, particularly psycho-behavioural approaches, may confer beneficial effects on CRF and depressive symptoms in patients with colorectal cancer. Notably, while intervention effects on CRF were observed across time points, results were consistent and displayed lower heterogeneity at medium-term and long-term follow-up, whereas short-term findings were highly variable. Moreover, effects on anxiety symptoms were not statistically significant. Given the very low to moderate certainty of evidence and substantial short-term heterogeneity, current findings are insufficient to determine the most effective intervention modalities or dosages. Further well‑designed, adequately powered RCTs are needed to confirm these effects and delineate the optimal characteristics of psychosocial interventions for reducing CRF in this population.

## Supplementary Information

Below is the link to the electronic supplementary material.

ESM 1 (DOCX 38.2 KB)

## Data Availability

No datasets were generated or analysed during the current study.
