# Supplementary material for: Effects of psychosocial interventions on cancer-related fatigue in patients with colorectal cancer: a systematic review and meta-analysis of randomised controlled trials
Source: Support Care Cancer. 2026 Mar 17;34(4):331. doi: 10.1007/s00520-026-10565-6 (PMC12992422; doi:10.1007/s00520-026-10565-6)
Supplement: Supplementary file 1 — (DOCX 38.2 KB) [file 520_2026_10565_MOESM1_ESM.docx]

**Supplementary Material A**

**Table 1.** Search strategy on databases.

| Database: Medline via OvidSP | | |
| --- | --- | --- |
| Literature searching date: 31/08/2025 | | |
| #1 | exp colorectal neoplasms/ [exp=explode] | 256770 |
| #2 | ((gastrointestinal or gastro-intestinal or "digestive tract" or colon or colonic or rectum or rectal or colorectal) and (neoplasm* or cancer* or tumor* or tumour* or carcinoma* or malignan* or metastas*)).mp. [mp=title, book title, abstract, original title, name of substance word, subject heading word, floating sub-heading word, keyword heading word, organism supplementary concept word, protocol supplementary concept word, rare disease supplementary concept word, unique identifier, synonyms, population supplementary concept word, anatomy supplementary concept word] | 433029 |
| #3 | #1 or #2 | 441774 |
| #4 | exp psychosocial intervention/ [exp=explode] | 1487 |
| #5 | exp psychology, social/ [exp=explode] | 1061586 |
| #6 | exp psychotherapy/ [exp=explode] | 232214 |
| #7 | exp counseling/ [exp=explode] | 51016 |
| #8 | ((psychological or psycho-social or psychotherap* or psychosocial* or psychoeduct* or psychodynamic or psychoanalyses or psycho* or cognitive or behavio* or mindfulness or "acceptance and commitment" or "attention and interpretation" or relax* or narrative or nondirective or socioenvironmental or reality or imagery or autogenic or stress or emotion* or social or interpersonal or individual or couple or marital or group or family or coping skill* or social skill* or motivat* or self control or self-control) and (therap* or intervention* or training or support or nursing or education or treatment or management)).mp. [mp=title, book title, abstract, original title, name of substance word, subject heading word, floating sub-heading word, keyword heading word, organism supplementary concept word, protocol supplementary concept word, rare disease supplementary concept word, unique identifier, synonyms, population supplementary concept word, anatomy supplementary concept word] | 6209839 |
| #9 | #4 or #5 or #6 or #7 or #8 | 6747927 |
| #10 | exp fatigue/ [exp=explode] | 41529 |
| #11 | (fatigue or cancer-related fatigue or cancer related fatigue or CRF or tired or tiredness or weary or weariness or exhaustion or exhausted or lackluster or apathy or apathetic or lassitude or weakness or lethargy or lethargic or asthenia or asthenic).mp. [mp=title, book title, abstract, original title, name of substance word, subject heading word, floating sub-heading word, keyword heading word, organism supplementary concept word, protocol supplementary concept word, rare disease supplementary concept word, unique identifier, synonyms, population supplementary concept word, anatomy supplementary concept word] | 265817 |
| #12 | ((lack or loss or lost) adj3 (energy or vigour or vitality)).mp. [mp=title, book title, abstract, original title, name of substance word, subject heading word, floating sub-heading word, keyword heading word, organism supplementary concept word, protocol supplementary concept word, rare disease supplementary concept word, unique identifier, synonyms, population supplementary concept word, anatomy supplementary concept word] | 6615 |
| #13 | (feel and (drained or sleepy or sleepiness or drowsy or drowsiness or sluggish)).mp. [mp=title, book title, abstract, original title, name of substance word, subject heading word, floating sub-heading word, keyword heading word, organism supplementary concept word, protocol supplementary concept word, rare disease supplementary concept word, unique identifier, synonyms, population supplementary concept word, anatomy supplementary concept word] | 162 |
| #14 | #10 or #11 or #12 or #13 | 271893 |
| #15 | exp randomized controlled trial/ or exp controlled clinical trial/ [exp=explode] | 733115 |
| #16 | (random* or RCT or placebo).mp. [mp=title, book title, abstract, original title, name of substance word, subject heading word, floating sub-heading word, keyword heading word, organism supplementary concept word, protocol supplementary concept word, rare disease supplementary concept word, unique identifier, synonyms, population supplementary concept word, anatomy supplementary concept word] | 1666254 |
| #17 | #15 or #16 | 1737222 |
| #18 | #3 and #9 and #14 and #17 | 442 |
| Database: EMBASE via OvidSP | | |
| Literature searching date: 31/08/2025 | | |
| #1 | exp colorectal tumor/ [exp=explode] | 525315 |
| #2 | ((gastrointestinal or gastro-intestinal or "digestive tract" or colon or colonic or rectum or rectal or colorectal) and (neoplasm* or cancer* or tumor* or tumour* or carcinoma* or malignan* or metastas*)).mp. [mp=title, abstract, heading word, drug trade name, original title, device manufacturer, drug manufacturer, device trade name, keyword heading word, floating subheading word, candidate term word] | 876660 |
| #3 | #1 or #2 | 890483 |
| #4 | exp psychosocial intervention/ [exp=explode] | 4663 |
| #5 | exp social psychology/ [exp=explode] | 163030 |
| #6 | exp psychotherapy/ [exp=explode] | 342275 |
| #7 | exp counseling/ [exp=explode] | 222283 |
| #8 | ((psychological or psycho-social or psychotherap* or psychosocial* or psychoeduct* or psychodynamic or psychoanalyses or psycho* or cognitive or behavio* or mindfulness or "acceptance and commitment" or "attention and interpretation" or relax* or narrative or nondirective or socioenvironmental or reality or imagery or autogenic or stress or emotion* or social or interpersonal or individual or couple or marital or group or family or coping skill* or social skill* or motivat* or self control or self-control) and (therap* or intervention* or training or support or nursing or education or treatment or management)).mp. [mp=title, abstract, heading word, drug trade name, original title, device manufacturer, drug manufacturer, device trade name, keyword heading word, floating subheading word, candidate term word] | 7220246 |
| #9 | #4 or #5 or #6 or #7 or #8 | 7426627 |
| #10 | exp fatigue/ [exp=explode] | 378148 |
| #11 | (fatigue or cancer-related fatigue or cancer related fatigue or CRF or tired or tiredness or weary or weariness or exhaustion or exhausted or lackluster or apathy or apathetic or lassitude or weakness or lethargy or lethargic or astenia or asthenic).mp. [mp=title, abstract, heading word, drug trade name, original title, device manufacturer, drug manufacturer, device trade name, keyword heading word, floating subheading word, candidate term word] | 729587 |
| #12 | ((lack or loss or lost) adj3 (energy or vigour or vitality)).mp. [mp=title, abstract, heading word, drug trade name, original title, device manufacturer, drug manufacturer, device trade name, keyword heading word, floating subheading word, candidate term word] | 14571 |
| #13 | (feel and (drained or sleepy or sleepiness or drowsy or drowsiness or sluggish)).mp. [mp=title, abstract, heading word, drug trade name, original title, device manufacturer, drug manufacturer, device trade name, keyword heading word, floating subheading word, candidate term word] | 540 |
| #14 | #10 or #11 or #12 or #13 | 750038 |
| #15 | exp randomized controlled trial/ or exp controlled clinical trial/ [exp=explode] | 1259799 |
| #16 | (random* or RCT or placebo).mp. [mp=title, abstract, heading word, drug trade name, original title, device manufacturer, drug manufacturer, device trade name, keyword heading word, floating subheading word, candidate term word] | 2988821 |
| #17 | #15 or #16 | 3148444 |
| #18 | #3 and #9 and #14 and #17 | 6636 |
| Database: Web of Science Core Collection | | |
| Literature searching date: 31/08/2025 | | |
| #1 | (gastrointestinal or gastro-intestinal or “digestive tract” or colon or colonic or rectum or rectal or colorectal) and (neoplasm* or cancer* or tumor* or tumour* or carcinoma* or malignan* or metastas*) (Topic) [Topic searches title, abstract, author keywords, and Keywords Plus.] | 565753 |
| #2 | (psychological or psycho-social or psychotherap* or psychosocial* or psychoeduct* or psychodynamic or psychoanalyses or psycho* or cognitive or behavio* or mindfulness or "acceptance and commitment" or "attention and interpretation" or relax* or narrative or nondirective or socioenvironmental or reality or imagery or autogenic or stress or emotion* or social or interpersonal or individual or couple or marital or group or family or coping skill* or social skill* or motivat* or self control or self-control) and (therap* or intervention* or training or support or nursing or education or treatment or management) (Topic) [Topic searches title, abstract, author keywords, and Keywords Plus.] | 7903355 |
| #3 | fatigue or cancer-related fatigue or cancer related fatigue or CRF or tired or tiredness or weary or weariness or exhaustion or exhausted or lackluster or apathy or apathetic or lassitude or weakness or lethargy or lethargic or astenia or asthenic (Topic) [Topic searches title, abstract, author keywords, and Keywords Plus.] | 796599 |
| #4 | (lack or loss or lost) and (energy or vigour or vitality) (Topic) [Topic searches title, abstract, author keywords, and Keywords Plus.] | 417372 |
| #5 | feel and (drained or sleepy or sleepiness or drowsy or drowsiness or sluggish) (Topic) [Topic searches title, abstract, author keywords, and Keywords Plus.] | 1669 |
| #6 | #3 or #4 or #5 | 1206152 |
| #7 | random* or RCT or placebo (Topic) [Topic searches title, abstract, author keywords, and Keywords Plus.] | 2959021 |
| #8 | #1 and #2 and #6 and #7 | 1132 |
| Database: CINAHL Ultimate via EBSCO | | |
| Literature searching date: 31/08/2025 | | |
| #1 | MH colorectal neoplasms [MH=exact subject heading] | 31393 |
| #2 | TI ((gastrointestinal or gastro-intestinal or “digestive tract” or colon or colonic or rectum or rectal or colorectal) and (neoplasm* or cancer* or tumor* or tumour* or carcinoma* or malignan* or metastas*)) or AB ((gastrointestinal or gastro-intestinal or “digestive tract”or colon or colonic or rectum or rectal or colorectal) and (neoplasm* or cancer* or tumor* or tumour* or carcinoma* or malignan* or metastas*)) [TI=title; AB=abstract] | 70273 |
| #3 | #1 or #2 | 76808 |
| #4 | MH psychosocial intervention [MH=exact subject heading] | 1720 |
| #5 | MH psychology, social [MH=exact subject heading] | 4195 |
| #6 | MH psychotherapy [MH=exact subject heading] | 25211 |
| #7 | MH counseling [MH=exact subject heading] | 36571 |
| #8 | TI ((psychological or psycho-social or psychotherap* or psychosocial* or psychoeduct* or psychodynamic or psychoanalyses or psycho* or cognitive or behavio* or mindfulness or "acceptance and commitment" or "attention and interpretation" or relax* or narrative or nondirective or socioenvironmental or reality or imagery or autogenic or stress or emotion* or social or interpersonal or individual or couple or marital or group or family or coping skill* or social skill* or motivat* or self control or self-control) and (therap* or intervention* or training or support or nursing or education or treatment or management)) or AB ((psychological or psycho-social or psychotherap* or psychosocial* or psychoeduct* or psychodynamic or psychoanalyses or psycho* or cognitive or behavio* or mindfulness or "acceptance and commitment" or "attention and interpretation" or relax* or narrative or nondirective or socioenvironmental or reality or imagery or autogenic or stress or emotion* or social or interpersonal or individual or couple or marital or group or family or coping skill* or social skill* or motivat* or self control or self-control) and (therap* or intervention* or training or support or nursing or education or treatment or management)) [TI=title; AB=abstract] | 1250526 |
| #9 | #4 or #5 or #6 or #7 or #8 | 1289314 |
| #10 | MH fatigue [MH=exact subject heading] | 23388 |
| #11 | TI (fatigue or cancer-related fatigue or cancer related fatigue or CRF or tired or tiredness or weary or weariness or exhaustion or exhausted or lackluster or apathy or apathetic or lassitude or weakness or lethargy or lethargic or astenia or asthenic) or AB (fatigue or cancer-related fatigue or cancer related fatigue or CRF or tired or tiredness or weary or weariness or exhaustion or exhausted or lackluster or apathy or apathetic or lassitude or weakness or lethargy or lethargic or astenia or asthenic) [TI=title; AB=abstract] | 90546 |
| #12 | TI ((lack or loss or lost) and (energy or vigour or vitality)) or AB ((lack or loss or lost) and (energy or vigour or vitality)) [TI=title; AB=abstract] | 9962 |
| #13 | TI (feel and (drained or sleepy or sleepiness or drowsy or drowsiness or sluggish)) or AB (feel and (drained or sleepy or sleepiness or drowsy or drowsiness or sluggish)) [TI=title; AB=abstract] | 69 |
| #14 | #10 or #11 or #12 or #13 | 106796 |
| #15 | PT randomized controlled trial or PT controlled clinical trial [PT=publication type] | 160529 |
| #16 | TI (random* or RCT or placebo) or AB (random* or RCT or placebo) [TI=title; AB=abstract] | 491885 |
| #17 | #15 or #16 | 517884 |
| #18 | #3 and #9 and #14 and #17 | 192 |
| Database: Cochrane Central Register of Controlled Trials | | |
| Literature searching date: 31/08/2025 | | |
| #1 | ((gastrointestinal or gastro-intestinal or “digestive tract” or colon or colonic or rectum or rectal or colorectal) and (neoplasm* or cancer* or tumor* or tumour* or carcinoma* or malignan* or metastas*)):ti,ab,kw [ti=title; ab=abstract; kw=keyword] | 43926 |
| #2 | ((psychological or psycho-social or psychotherap* or psychosocial* or psychoeduct* or psychodynamic or psychoanalyses or psycho* or cognitive or behavio* or mindfulness or "acceptance and commitment" or "attention and interpretation" or relax* or narrative or nondirective or socioenvironmental or reality or imagery or autogenic or stress or emotion* or social or interpersonal or individual or couple or marital or group or family or coping skill* or social skill* or motivat* or self control or self-control) and (therap* or intervention* or training or support or nursing or education or treatment or management)):ti,ab,kw [ti=title; ab=abstract; kw=keyword] | 1174183 |
| #3 | (fatigue or cancer-related fatigue or cancer related fatigue or CRF or tired or tiredness or weary or weariness or exhaustion or exhausted or lackluster or apathy or apathetic or lassitude or weakness or lethargy or lethargic or astenia or asthenic):ti,ab,kw [ti=title; ab=abstract; kw=keyword] | 77941 |
| #4 | (lack or loss or lost) and (energy or vigour or vitality):ti,ab,kw [ti=title; ab=abstract; kw=keyword] | 15122 |
| #5 | feel and (drained or sleepy or sleepiness or drowsy or drowsiness or sluggish):ti,ab,kw [ti=title; ab=abstract; kw=keyword] | 610 |
| #6 | #3 or #4 or #5 | 92013 |
| #7 | random* or RCT or placebo:ti,ab,kw [ti=title; ab=abstract; kw=keyword] | 1514833 |
| #8 | #1 and #2 and #6 and #7 | 3112 |
| Database: APA PsycInfo via OvidSP | | |
| Literature searching date: 31/08/2025 | | |
| #1 | exp colorectal neoplasms/ [exp=explode] | 2234 |
| #2 | ((gastrointestinal or gastro-intestinal or "digestive tract" or colon or colonic or rectum or rectal or colorectal) and (neoplasm* or cancer* or tumor* or tumour* or carcinoma* or malignan* or metastas*)).mp. [mp=title, abstract, heading word, table of contents, key concepts, original title, tests & measures, mesh word] | 6918 |
| #3 | #1 or #2 | 6918 |
| #4 | exp psychosocial interventions/ [exp=explode] | 2342 |
| #5 | exp social psychology/ [exp=explode] | 17480 |
| #6 | exp psychotherapy/ [exp=explode] | 234366 |
| #7 | exp counseling/ [exp=explode] | 87612 |
| #8 | ((psychological or psycho-social or psychotherap* or psychosocial* or psychoeduct* or psychodynamic or psychoanalyses or psycho* or cognitive or behavio* or mindfulness or "acceptance and commitment" or "attention and interpretation" or relax* or narrative or nondirective or socioenvironmental or reality or imagery or autogenic or stress or emotion* or social or interpersonal or individual or couple or marital or group or family or coping skill* or social skill* or motivat* or self control or self-control) and (therap* or intervention* or training or support or nursing or education or treatment or management)).mp. [mp=title, abstract, heading word, table of contents, key concepts, original title, tests & measures, mesh word] | 2050431 |
| #9 | #4 or #5 or #6 or #7 or #8 | 2142164 |
| #10 | exp fatigue/ [exp=explode] | 13128 |
| #11 | (fatigue or cancer-related fatigue or cancer related fatigue or CRF or tired or tiredness or weary or weariness or exhaustion or exhausted or lackluster or apathy or apathetic or lassitude or weakness or lethargy or lethargic or astenia or asthenic).mp. [mp=title, abstract, heading word, table of contents, key concepts, original title, tests & measures, mesh word] | 74291 |
| #12 | ((lack or loss or lost) adj3 (energy or vigour or vitality)).mp. [mp=title, abstract, heading word, table of contents, key concepts, original title, tests & measures, mesh word] | 1144 |
| #13 | (feel and (drained or sleepy or sleepiness or drowsy or drowsiness or sluggish)).mp. [mp=title, abstract, heading word, table of contents, key concepts, original title, tests & measures, mesh word] | 114 |
| #14 | #10 or #11 or #12 or #13 | 75155 |
| #15 | exp randomized controlled trial/ [exp=explode] | 1784 |
| #16 | (random* or RCT or placebo).mp. [mp=title, abstract, heading word, table of contents, key concepts, original title, tests & measures, mesh word] | 301743 |
| #17 | #15 or #16 | 301743 |
| #18 | #3 and #9 and #14 and #17 | 63 |
| Database: WanFang Data | | |
| Literature searching date: 31/08/2025 | | |
| #1 | 主题:(消化道肿瘤 or 消化系统肿瘤 or 胃肠道肿瘤 or 胃肠肿瘤 or肠癌 or 大肠癌 or 大肠肿瘤 or 结肠癌 or 结肠肿瘤 or 直肠癌 or 直肠肿瘤 or 结直肠癌 or 结直肠肿瘤) | 10834 |
| #2 | 主题: (心理* or 社会 or 教育 or 认知 or 行为 or 正念 or 接纳与承诺 or关注和解释 or放松 or 叙事 or 压力 or 情绪 or 应对 or 个体 or 同伴 or 夫妻 or 伴侣 or 家庭 or动机 or 自我控制) and 主题: (疗法 or 干预 or 支持 or 护理 or 治疗) | 100743 |
| #3 | 主题:(疲劳 or 疲乏 or 疲倦 or 癌因性疲乏) | 12853 |
| #4 | 主题: (缺乏 or 丧失) and (活力 or 生命力) | 1425 |
| #5 | 主题: (困倦 or 精疲力竭 or 昏昏欲睡) | 267 |
| #6 | #3 or #4 or #5 | 12934 |
| #7 | 主题:(随机对照 or RCT) | 108324 |
| #8 | #1 and #2 and #6 and #7 | 201 |
| Database: China National Knowledge Infrastructure (CNKI) | | |
| Literature searching date: 31/08/2025 | | |
| #1 | 主题=消化道肿瘤 or 主题=消化系统肿瘤 or 主题=胃肠道肿瘤 or 主题=胃肠肿瘤 or 主题=肠癌 or 主题=大肠癌 or 主题=大肠肿瘤 or 主题=结肠癌 or 主题=结肠肿瘤 or 主题=直肠癌 or 主题=直肠肿瘤 or 主题=结直肠癌 or 主题=结直肠肿瘤 | 11823 |
| #2 | (主题=心理* or 主题=社会 or 主题=教育 or 主题=认知 or 主题=行为 or 主题=正念 or 主题=接纳与承诺 or 主题=关注和解释 or 主题=放松 or 主题=叙事 or 主题=压力 or 主题=情绪 or 主题=应对 or 主题=个体 or 主题=同伴 or 主题=夫妻 or 主题=伴侣 or 主题=家庭 or 主题=动机 or 主题=自我控制) and (主题=疗法 or 主题=干预 or 主题=支持 or 主题=护理 or 主题=治疗) | 102561 |
| #3 | 主题=疲劳 or主题=疲乏 or主题=疲倦 or主题=癌因性疲乏 | 17156 |
| #4 | (主题=缺乏 or主题=丧失) and (主题=活力 or主题=生命力) | 934 |
| #5 | 主题=困倦 or主题=精疲力竭 or主题=昏昏欲睡 | 164 |
| #6 | #3 or #4 or #5 | 17971 |
| #7 | 主题=随机对照 or主题=RCT | 47134 |
| #8 | #1 and #2 and #6 and #7 | 62 |
